# LncRNA ZFAS1 regulates ATIC transcription and promotes the proliferation and migration of hepatocellular carcinoma through the PI3K/AKT signaling pathway

**DOI:** 10.1007/s00432-024-05877-1

**Published:** 2024-07-13

**Authors:** Baoyang Luo, Lin Zhuang, Ju Huang, Longqing Shi, Li Zhang, Maoqun Zhu, Yunjie Lu, Qiang Zhu, Donglin Sun, Hao Wang, Haisheng Fang

**Affiliations:** 1 Department of Hepatobiliary and Pancreatic Surgery, Third Affiliated Hospital of Soochow University, 185th Juqian Street, Changzhou, Jiangsu 213003 China; 2 Department of Hepatobiliary and Pancreatic Surgery, The Affiliated Taizhou People’s Hospital of Nanjing Medical University, Taizhou, Jiangsu 225300 China; 3 Department of General Surgery, Wujin Affiliated Hospital of Jiangsu University and The Wujin Clinical college of Xuzhou Medical University, Changzhou, Jiangsu 213000 China; 4 School of Pharmacy, Nantong University, Nantong, Jiangsu 226001 China; 5 Department of General Surgery, Children’s Hospital of Nanjing Medical University, Nanjing, 210008 China; 6 Department of Pathology, The First Affiliated Hospital with Nanjing Medical University, Nanjing, 210029 China

**Keywords:** Hepatocellular carcinoma, lncRNA ZFAS1, ATIC, SC79, PI3K/AKT signaling pathway

## Abstract

**Purpose:**

Long noncoding RNAs (lncRNAs) exert a significant influence on various cancer-related processes through their intricate interactions with RNAs. Among these, lncRNA ZFAS1 has been implicated in oncogenic roles in multiple cancer types. Nevertheless, the intricate biological significance and underlying mechanism of ZFAS1 in the initiation and progression of hepatocellular carcinoma (HCC) remain largely unexplored.

**Methods:**

Analysis of The Cancer Genome Atlas Program (TCGA) database revealed a notable upregulation of lncRNA ZFAS1 in HCC tissues. To explore its function, we investigated colony formation and performed CCK-8 assays to gauge cellular proliferation and wound healing, Transwell assays to assess cellular migration, and an in vivo study employing a nude mouse model to scrutinize tumor growth and metastasis. Luciferase reporter assay was used to confirm the implicated interactions. Rescue experiments were conducted to unravel the plausible mechanism underlying the activation of the PI3K/AKT pathway by lncRNAs ZFAS1 and ATIC.

**Results:**

ZFAS1 and ATIC were significantly upregulated in the HCC tissues and cells. ZFAS1 knockdown inhibited cell proliferation and migration. We observed a direct interaction between the lncRNA ZFAS1 and ATIC. ATIC knockdown also suppressed cell proliferation and migration. SC79, an activator of AKT, partially restores the effects of lncRNA ZFAS1/ATIC knockdown on cell proliferation and migration. Knockdown of lncRNA ZFAS1/ATIC inhibited tumor growth and lung metastasis in vivo.

**Conclusion:**

Overall, lncRNA ZFAS1 regulates ATIC transcription and contributes to the growth and migration of HCC cells through the PI3K/AKT signaling pathway.

**Supplementary Information:**

The online version contains supplementary material available at 10.1007/s00432-024-05877-1.

## Introduction

Hepatocellular carcinoma (HCC) is a highly prevalent neoplasm that significantly contributes to cancer-related mortality worldwide (Siegel et al. 2017). Despite remarkable advancements in diagnostic techniques and surgical procedures, and the advent of novel molecular targeted therapies, HCC remains the primary cause of cancer-related fatalities globally, accounting for a staggering 600 thousand annual deaths (Ma et al. 2017; Omata et al. 2017). Unfortunately, the current surveillance and treatment approaches fail to meet satisfactory standards. A substantial risk factor affecting the prognosis of HCC is the high incidence of tumor recurrence and distant metastasis after hepatectomy (Pi et al. 2016). Therefore, it is imperative to elucidate the mechanisms underlying the initiation and metastasis of HCC to enhance its early detection and prognostic capabilities.

Long noncoding RNAs (lncRNAs) are RNA molecules that exceed 200 bp and do not possess the capacity to encode proteins (Fang et al. 2016). LncRNAs play a crucial role in diverse biological processes including autophagy (Liang et al. 2020), proliferation (Hu et al. 2017; Luo et al. 2019) and metastasis (Abbastabar et al. 2018) of cancer cells. Furthermore, lncRNAs orchestrate tumorigenesis and metastasis in diverse malignancies, including HCC, by perturbing gene expression at the transcriptional or epigenetic level (Abbastabar et al. 2018; Yao et al. 2022). Multiple lncRNAs have been reported to regulate lung cancer growth and metastasis (Huang et al. 2021b, 2024). The lncRNA ANCR exhibited heightened expression in both HCC tissues and cells, displaying a positive association with tumor differentiation, TNM stage, tumor size, and the presence of portal vein tumor thrombus (Wen et al. 2020). Similarly, the lncRNA ALKBH3-AS1 displays heightened expression in HCC, with its elevated levels notably linked to reduced survival among HCC patients (Lu et al. 2022). Another newly discovered lncRNA, ZFAS1, has been found to exhibits increased expression in diverse human malignancies, including colorectal cancer (Wang et al. 2022), HCC (Zhu et al. 2023a), pancreatic cancer (Zhuo et al. 2023) and laryngeal cancer (Lu et al. 2023). The lncRNA ZFAS1 contributes to the progression of this disease through the miR-624/MDK/JNK/ERK/P38 pathway (Duan et al. 2020a). However, the exact biological function of ZFAS1 and its underlying molecular mechanisms, particularly in HCC, remain largely unknown.

In this study, we analyzed the expression of disease-associated lncRNAs in HCC tissues. Remarkably, we discovered that lncRNA ZFAS1 exhibited an upregulated expression pattern, specifically within HCC tissues. Subsequently, we investigated the effects of ZFAS1 expression on crucial cellular processes. Moreover, we explored the potential interactions between the lncRNA ZFAS1 and the gene to elucidate their involvement in the functional mechanisms of ZFAS1.

## Materials and methods

### Bioinformatics analysis

RNA-Seq data were obtained from the Cancer Genome Atlas (TCGA) database, encompassing a comprehensive set of 50 normal samples and 373 HCC samples. Differential expression analysis was conducted using the limma package in R4.1.2, employing a stringent threshold of FDR < 0.05 and |log_2_FC|>1 to identify significantly differentially expressed genes (DEGs) and lncRNAs (DE-lncRNAs). To illustrate the differences in gene expression between tumor and control samples, we utilized the Pheatmap version 1.0.8 package in R4.1.2, to perform bidirectional hierarchical clustering according to the expression profiles of DE-lncRNAs and DEGs. The Pearson Correlation Coefficient (PCC) between DE-lncRNAs and DEGs was computed using the COR function in R4.1.2 language. Subsequently, by selecting connection pairs with a PCC > 0.6 and *P* < 0.05, we constructed and visualized a co-expression network of DE-lncRNAs and DEGs using Cytoscape version 3.6.1.

Furthermore, patients with HCC were categorized into high- and low-expression groups of lncRNA ZFAS1 based on their expression levels. Fisher’s exact test was used to assess the clinical relationship between ZFAS1 expression and age, sex, neoplasm histologic grade, pathologic M, pathologic N, pathologic T, pathologic stage, recurrence, and vital status.

### Cell culture

The HepG2, Hep3B, and WRL68 cell lines derived from human hepatocellular carcinoma, were acquired from the Cell Bank of the Institute of Biochemistry and Cell Biology (Chinese Academy of Sciences, Shanghai, China). The cells were cultured in Modified Eagle Medium (MEM) (Servicebio, China) supplemented with 1% penicillin/streptomycin (Gibco, USA) and 10% fetal bovine serum (FBS) (Gibco, USA) at 37 °C in a humidified incubator with 5% CO_2_.

### Patient samples collection

Five paired HCC tissue samples and their corresponding adjacent normal tissues were obtained from patients who underwent surgical resection between February 2022 and May 2022 at our hospital. Resected tissue samples were immediately frozen in liquid nitrogen and stored at -80 °C before RNA extraction. This study was approved by the Ethics Committee of the Affiliated Taizhou People’s Hospital of Nanjing Medical University, and written informed consent was obtained from all patients.

### Cell transfection

Small interfering RNA (siRNA) molecules designed to target lncRNA ZFAS1 (si-lncRNA ZFAS1) and their corresponding control (si-NC), as well as siRNA targeting ATIC (si-ATIC) and its matched control (si-NC), were synthesized by Shanghai Generay Biotech Co., Ltd. (Shanghai, China). Prior to transfection, cells were cultured in a complete medium for a minimum of 24 h. For cell transfection, Lipofectamine 2000 (Thermo Fisher Scientific) was used in accordance with the manufacturer’s instructions to ensure efficient delivery of siRNA molecules into the cells.

### Real time-polymerase chain reaction (RT-PCR)

TRIzol reagent (Thermo Fisher Scientific) was used to extract total RNA from the cells. Subsequently, the extracted total RNA was reverse-transcribed using a PrimeScript RT Reagent Kit (Takara, Japan) following the manufacturer’s instructions. RT-qPCR was performed using a SYBR Green PCR Kit (Thermo Fisher Scientific). The RT-qPCR program commenced with an initial step at 95 °C for 2 min, succeeded by 40 cycles of denaturation at 95 °C for 15 s and annealing at 60 °C for 60 s. The expression levels of the target genes were normalized to the reference gene GAPDH and calculated utilizing the 2^−ΔΔCT^ method. The primer sequences used in this study are listed in Table 1.

**Table 1:** The primer sequences used in this study

Name	Primers
GAPDH-hF	TGACAACTTTGGTATCGTGGAAGG
GAPDH-hR	AGGCAGGGATGATGTTCTGGAGAG
lnc-ZFAS1-hF	TGTGCTGCTCGAGACTACATTT
lnc-ZFAS1-hR	TTCCAAAATCCATTCTGTACCC
ATIC-hF	AAGGCAAACTATTGGTGGCTTA
ATIC-hR	CAGTTTCTCAACCCATTCCTTC

### CCK-8 assay

Cell viability was assessed using the CCK-8 assay (Beyotime, China). Transfected cells were resuspended at a concentration of 1 × 10^4^ cells/mL and plated in 96-well plates. Following 24, 48, and 72 h of incubation, the cells were treated with CCK-8 (10 µL) for 2 h. Absorbance at 450 nm was measured using a microplate reader.

### Colony formation assay

The cells were seeded into a 6-well plate and subjected to a two-week cultivation until discernible cell colonies became visible to the naked eye. Subsequently, the cells were rinsed and fixed in 4% paraformaldehyde solution for 10 min. Following fixation, staining was performed using 1% crystal violet solution (Beyotime, China) for 10 min. After drying, the colonies were counted under a microscope to determine the colony formation rate.

### Transwell migration assay

Cell migration was investigated using transwell chambers (Guangzhou Jet Bio-Filtration Co., Ltd., China). Briefly, cells subjected to different treatments were seeded in the upper compartment of the transwell chambers, while the lower compartment was supplemented with complete medium. After 48 h of incubation at 37 °C, the cells were immobilized in a 4% paraformaldehyde solution for 20 min. Subsequently, the cells were stained with 1% crystal violet for 20 min. Cell migration was visualized under a microscope, and the number of migrated cells was quantified.

### Wound healing assay

Migratory potential was evaluated using a wound healing assay. Initially, a marker pen was used to delineate a straight line on the surface of the 6-well plates. The transfected cells were then seeded in these plates and cultured until they reached 80–90% confluence. Subsequently, two parallel lines were inscribed perpendicularly to the initial marking line using a pipette tip. The scratched cells were then washed with PBS, and each well was filled with serum-free medium. Images of the cellular landscape were captured at the initiation of the assay and after a 48-hour duration, utilizing an inverted microscope.

### Dual-luciferase reporter assay

The cells were carefully distributed in a 96-well plate. At 48 h post-transfection, luciferase activity was quantified using a dual-luciferase reporter gene assay kit (Beyotime) according to the guidelines provided by the manufacturer’s instructions. To ensure consistency and accuracy, the data obtained were normalized by calculating the ratio of firefly to Renilla luciferase activity.

### Western blot analysis

RIPA buffer (Beyotime, China) was used to lyse the total proteins extracted from HCC cells. The BCA Protein Assay Kit (Thermo Fisher Scientific, USA) was used to determine protein concentration. Subsequently, lysate aliquots were subjected to sodium dodecyl sulfate-polyacrylamide gel electrophoresis and transferred electrophoretically onto polyvinylidene fluoride membranes. The membranes were subsequently obstructed in 5% skim milk, followed by overnight incubation with primary antibodies at a temperature of 4 °C. The following primary antibodies were used: anti-ATIC (10726-1-AP, 1:2000), anti-AKT (60203-2-Ig, 1:5000), anti-p-AKT (66444-1-Ig, 1:5000), anti-PI3K (20584-1-AP, 1:800), anti-p-PI3K (ab182651, 1:500), and anti-GAPDH (10494-1-AP, 1:10000). Afterward, the PVDF membrane was subjected to three washes with Tris Buffered Saline Tween, succeeded by a period of incubation with horseradish peroxidase (HRP)-conjugated secondary antibodies (Jackson ImmunoResearch, 1:5000) at 37 °C for 2 h. Visualization of the protein bands was achieved using an enhanced chemiluminescence kit (Beyotime, China), and the grayscale density of the bands was quantified using ImageJ software.

### Animal experiments

The animal experimental protocols were approved by the Committee for Ethical Review of Research of the Affiliated Wujin People’s Hospital of Jiangsu University (Protocol Number: SYXK 2018-0053). A total of nine male BALB/c-nu/nu nude mice, aged 4–6 weeks, were acquired from Shanghai SLAC Laboratory Animal Co., Ltd. (Shanghai, China). Following transfection with si-NC, si-lncRNA ZFAS1, or si-ATIC, cells were thoroughly washed with PBS. Subsequently, transfected cells were suspended at a density of 5 × 10^7^ cells/ml. The mice were then randomly assigned to three groups and injected with 100 µl of cell suspension into the forelimb through the axilla. The weight of the mice and length of the tumors were assessed weekly. After 4 weeks, the nude mice were humanely euthanized, and the bioluminescence emitted from the mouse body was collected using an IVIS Lumina XRMS In Vivo Imaging System Series III (PerkinElmer). The tumors were carefully peeled off and weighed. Tumor and lung tissues were collected and meticulously preserved in 4% paraformaldehyde solution for fixation.

### Hematoxylin and eosin (H&E) staining

Paraformaldehyde-fixed tissues were dehydrated through a succession of alcohol gradients and subsequently embedded in paraffin. Subsequently, paraffin-embedded sections were deparaffinized using xylene and rehydrated using an ethanol gradient. 10-min staining procedure with hematoxylin ensued, followed by immersion in 1% hydrochloric acid alcohol solution. The sections were then stained with an aqueous solution of eosin for 90 s, dehydrated using a series of alcohol gradients, and rendered transparent with xylene.

### Immunofluorescence

The tumor and lung tissues were fixed in 4% paraformaldehyde, followed by dehydration with sucrose. Subsequently, the tissues were embedded in optimal cutting temperature (OCT) compound and cryopreserved at -80 °C for 1 h. Then, they were sectioned into 10 μm thick sections. The frozen sections were baked in a 37 °C oven for 20 min to remove excess moisture. After drying, the sections were mounted with an anti-fluorescence quenching mounting medium (containing DAPI) and covered with a coverslip. Observations and photographs were obtained using a confocal laser microscope.

### Immunohistochemistry (IHC)

For IHC staining, paraffin-embedded tissue sections were subjected to antigen retrieval using citrate buffer, and the activity of endogenous peroxidase was inhibited using a 3% hydrogen peroxide solution. Subsequently, the sections were rinsed with phosphate-buffered saline and incubated with a primary antibody against ATIC (1:100 dilution, Proteintech, USA) for 1 h at 37 °C. Subsequently, the sections were subjected to a 30-min incubation with a secondary antibody labeled with horseradish peroxidase (HRP), followed by visualization using amine nickel sulfate-enhanced 3,3’-diaminobenzidine. The nuclei were stained with hematoxylin, and images were taken under a light microscope at 200x magnification.

### Statistical analysis

The data were subjected to analysis using GraphPad Prism 8.0 software and were expressed as the mean ± standard deviation (SD). T-tests were used to assess the differences between two groups, whereas one-way analysis of variance (ANOVA) was used to compare the differences among three or more groups. A significance level of *P* < 0.05 was considered statistically significant.

## Results

### Bioinformatics prediction of lncRNA ZFAS1 and targeting mRNA and validation

**Fig. 1:**
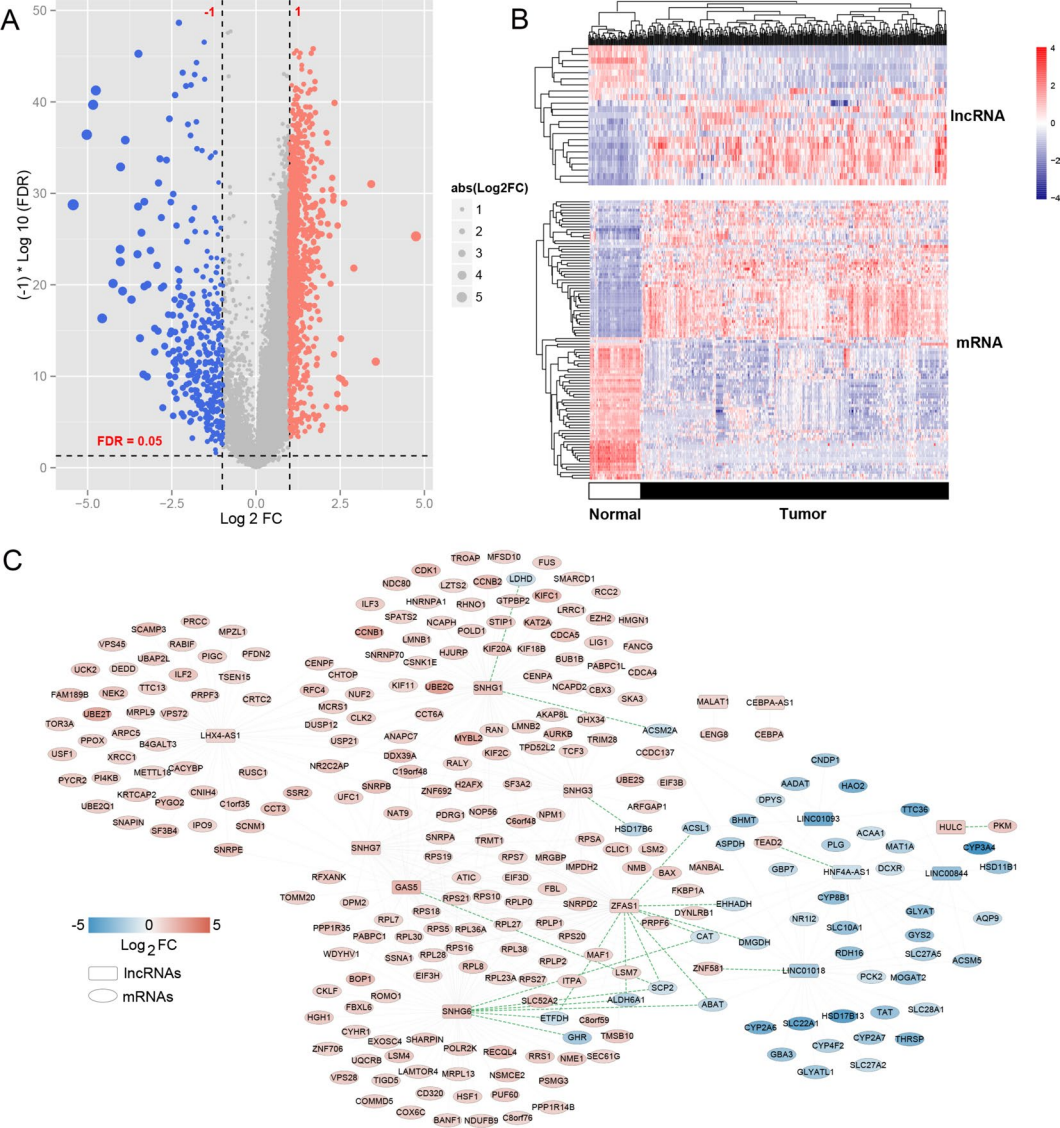
Expression of long non-coding RNA (lncRNA) ZFAS1 and ATIC in hepatocellular carcinoma (HCC). (**A**) Volcano plot of DE-lncRNAs and DEGs in the cancer genome atlas (TCGA) database. (**B**) Clustering heat map of DE-lncRNAs and DEGs in the TCGA database. (**C**) A DE-lncRNAs - DEGs co-expression network

**Fig. 2:**
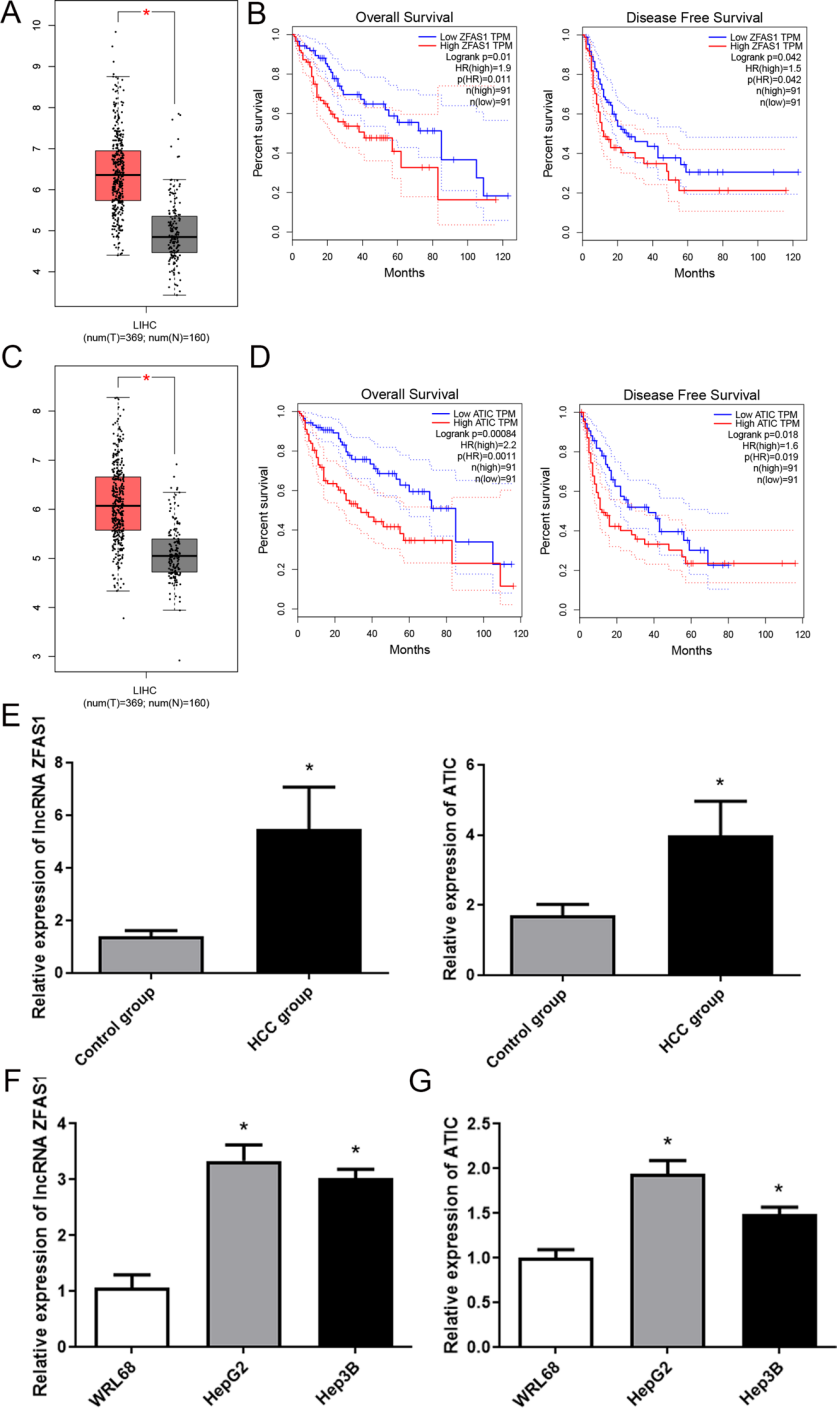
Long non-coding RNA (lncRNA) ZFAS1 and ATIC expression increased in hepatocellular carcinoma (HCC) cell lines. (**A**) Expression of lncRNA ZFAS1 analyzed in 369 HCC tissues and 160 normal samples by using the cancer genome atlas (TCGA) database. (**B**) Survival analysis of patients in TCGA according to lncRNA ZFAS1 expression. (**C**) Expression of ATIC analyzed in 369 HCC tissues and 160 normal samples by using TCGA database. (**D**) Survival analysis of patients in TCGA according to ATIC expression. (**E**) Expression of lncRNA ZFAS1 and ATIC in HCC and control tissues detected via RT-RCR. *P < 0.05, compared with control group. RT-RCR was applied to detect the relative expression of lncRNA ZFAS1 (**F**) and ATIC (**G**) in HCC cell lines (HepG2, Hep3B, and WRL68). *P < 0.05, compared with WRL68

Initially, we identified 1387 differentially expressed RNAs between HCC and control tissues using TCGA dataset. Among these, there were 24 DE-lncRNAs with 9 downregulated and 15 upregulated genes and 1363 DEGs with 383 downregulated genes and 980 upregulated genes (Fig. 1A). Hierarchical clustering analysis revealed systematic variations in lncRNA expression patterns between HCC and control tissues (Fig. 1B). Subsequently, using a predefined threshold, we identified 450 regulatory pairs co-expressed between the DE lncRNAs and DEGs. Based on these regulatory pairs, we established a co-expression network comprising 14 DE lncRNAs and 257 DEGs (Fig. 1C). Among them, lncRNA ZFAS1 exhibited significantly higher expression levels in HCC tissues than in control tissues (Fig. 2A), and lncRNA ZFAS1 overexpression was linked to poor disease-free survival and poor overall survival in HCC patients (Fig. 2B). LncRNA ZFAS1 is upregulated in HCC and is linked to the development and progression of the disease (Abozeid et al. 2022; Duan et al. 2020b; Zhou et al. 2019). Therefore, we selected lncRNA ZFAS1 as the target of our study. In addition, we found that the lncRNA ZFAS1 interacted with ATIC in the ceRNA network and that ATIC was highly expressed in HCC tissues (Fig. 2C). Furthermore, ATIC overexpression was linked to poor disease-free survival and overall survival in patients with HCC (Fig. 2D). To validate these findings, we used RT-PCR to assess the expression levels of ZFAS1 and ATIC in HCC and control tissues. The results demonstrated a notable upregulation of both lncRNA ZFAS1 and ATIC in HCC tissues compared to control tissues (Fig. 2E, *P* < 0.05). Consequently, we hypothesized that lncRNA ZFAS1 plays a crucial role in promoting HCC progression through its interaction with ATIC.

### Relationship between lncRNA ZFAS1 expression and clinical factors

Subsequently, the relationship between lncRNA ZFAS1 expression and clinical factors was analyzed. The results indicated that compared to the low lncRNA ZFAS1 expression group, the high lncRNA ZFAS1 expression group had a higher proportion of patients with neoplasm histologic grade 3, neoplasm histologic grade 4, and mortality, whereas fewer patients had neoplasm histologic grades 3 and 4 (Table 2, *P* < 0.05). However, no significant differences were observed between the two groups in terms of age, sex, pathologic M, pathologic N, pathologic T, pathologic stage, or recurrence (Table 2; *P* > 0.05).

**Table 2:** Relationship between ZFAS1 expression and clinical factors

characteristics total cases	*N* of case 373	ZFAS1 expression gorup		*P* value (fisher’s exact test)
		Low(*N* = 186)	High(*N* = 187)	
Age(years)				
≤ 60	177	84	93	4.063E-01
> 65	195	102	93	
NA	1	0	1	
Geneder				
Male	252	125	127	9.122E-01
Female	121	61	60	
Neoplasm histologic grade				
G1	55	37	18	3.395E-05
G2	178	101	77	
G3	123	44	79	
G4	12	3	9	
NA	5	1	4	
Pathologic M				
M0	267	122	145	6.287E-01
M1	4	1	3	
NA	102	63	39	
Pathologic N				
N0	253	117	136	1.281E-01
N1	4	0	4	
NA	116	69	47	
Pathologic T				
1	182	100	82	2.032E-01
2	95	45	50	
3	80	33	47	
4	13	6	7	
NA	3	2	1	
Pathologic stage				
1	172	93	79	1.401E-01
2	87	41	46	
3	85	35	50	
4	5	1	4	
NA	24	16	8	
Recurrence				
Yes	141	67	74	2.162E-01
No	179	98	81	
NA	53	21	32	
Vital status				
Dead	130	57	73	4.672E-02
Alive	237	128	109	
NA	6	1	5	

NA denotes samples for which no corresponding information was provided

### LncRNA ZFAS1 and ATIC expression increased in HCC cell lines

ZFAS1 and ATIC expression levels were assessed in various HCC cell lines (Hep3B, HepG2, and WRL68). Remarkably, lncRNAs ZFAS1 and ATIC exhibited pronounced upregulation in HepG2 and Hep3B cells compared to WRL68 cells, with HepG2 cells demonstrating particularly elevated expression levels (Fig. 2F, G, *P* < 0.05). Consequently, HepG2 cells were selected for subsequent experiments.

### Knockdown of lncRNA ZFAS1 inhibited proliferation and migration of HCC cells

**Fig. 3:**
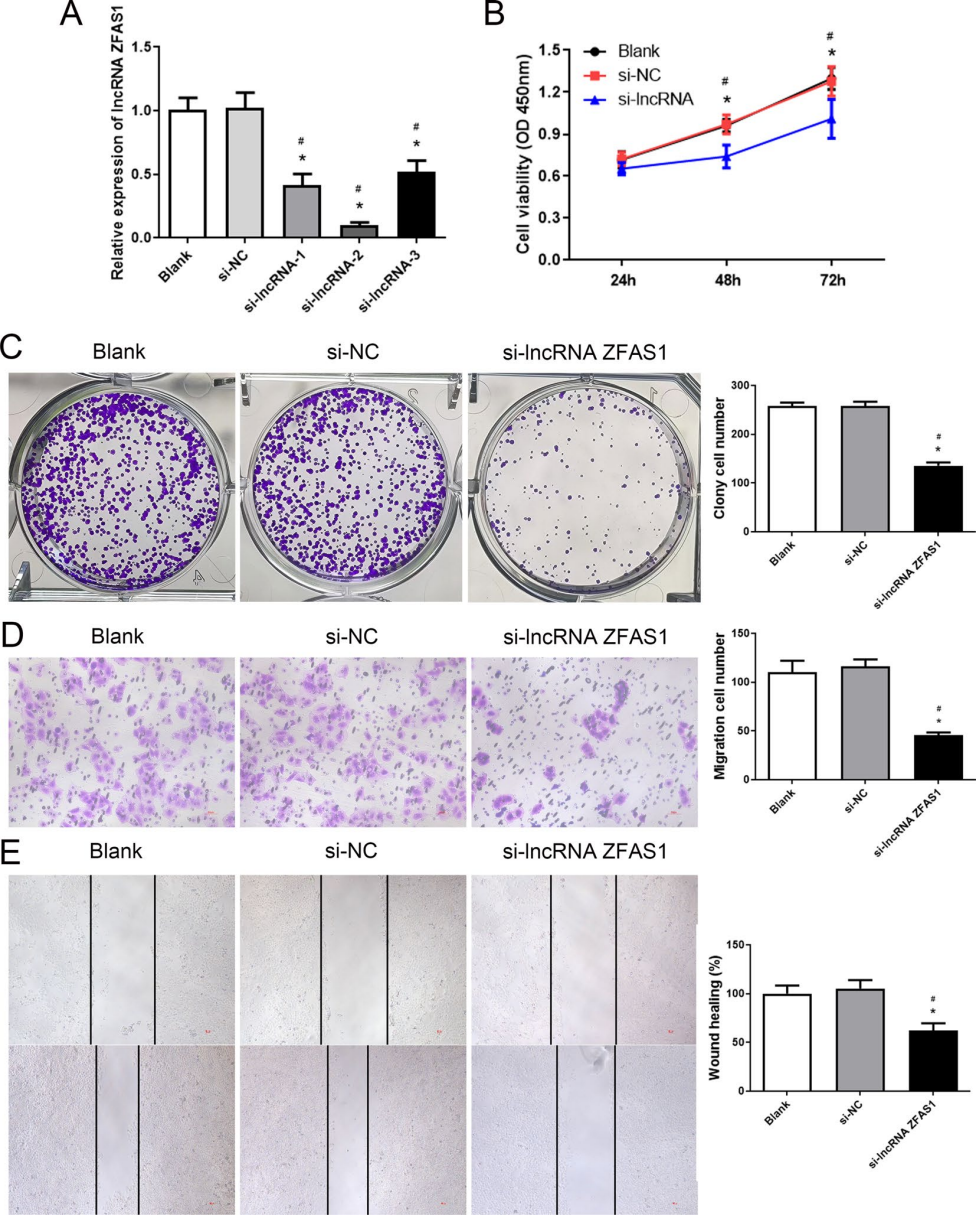
Knockdown of long non-coding RNA (lncRNA) ZFAS1 regulates the proliferation and migration of hepatocellular carcinoma (HCC) cells. (**A**) RT-PCR analysis of lncRNA ZFAS1 expression in HCC cells transfected with control (scrambled), si-lncRNA ZFAS1-1, si-lncRNA ZFAS1-2, and si-lncRNA ZFAS1-3. (**B**) CCK-8 assays showing the viability of HCC cells treated with si-lncRNA ZFAS1. (**C**) Colony formation assays showing the proliferation of si-lncRNA ZFAS1-transfected HCC cells and colonies were counted and captured. (**D**) Transwell assays showing the migration of HCC cells treated with si-lncRNA ZFAS1. (**E**) Wound healing assays showing the migration of HCC cells treated with si-lncRNA ZFAS1. *P < 0.05, compared with Blank group. ^#^*P* < 0.05, compared with si-NC group

To study the impact of lncRNA ZFAS1 on HCC cells, we knocked down lncRNA ZFAS. The knockdown efficiency was assessed using RT-PCR, and it was observed that the three siRNAs effectively downregulated lncRNA ZFAS1, with si-lncRNA ZFAS1-2 exhibiting the highest knockdown efficiency (Fig. 3A). Subsequently, a series of assays were performed to evaluate the cell proliferation and migration capabilities of HCC cells. CCK-8 assays indicated that si-lncRNA ZFAS1 significantly inhibited HepG2 cell viability (Fig. 3B). Similarly, colony formation assays revealed that si-lncRNA ZFAS1 markedly suppressed the clonogenic potential of HepG2 cells (Fig. 3C). Moreover, transwell and wound healing assays indicated that si-lncRNA ZFAS1 notably inhibited the migratory capacity of the cells (Fig. 3D, E). These results collectively implied that knockdown of lncRNA ZFAS1 inhibited the growth and migration of HCC cells.

### ATIC participated in the process of lncRNA ZFAS1 mediated proliferation and migration

**Fig. 4:**
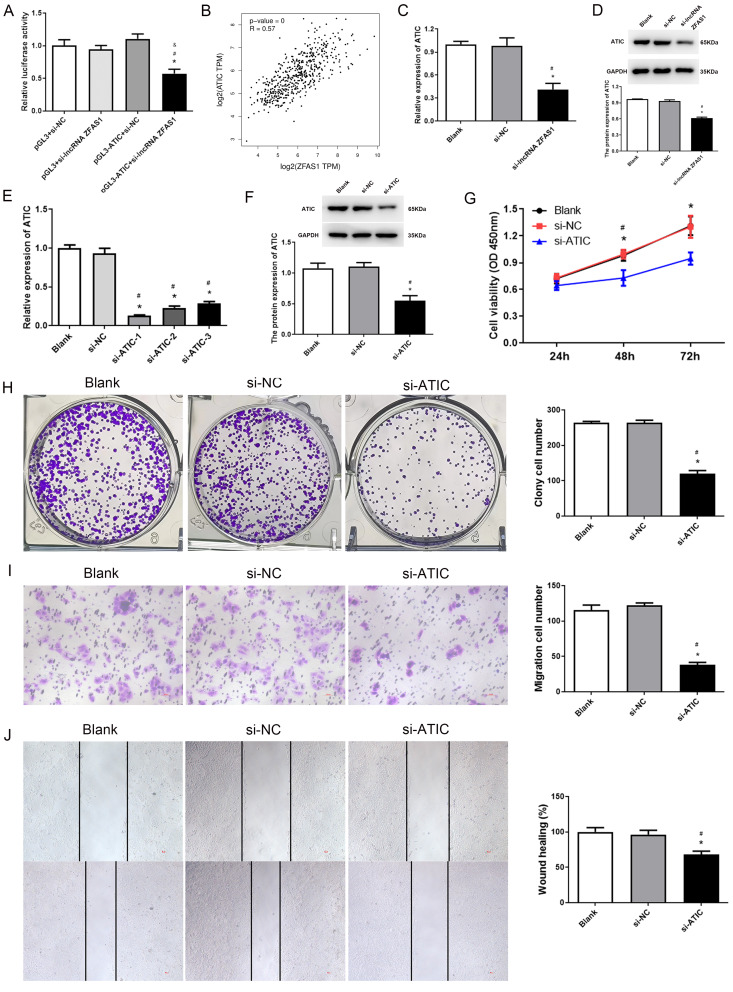
Knockdown of ATIC regulated proliferation and migration of hepatocellular carcinoma (HCC) cells. (**A**) Bioinformatic analysis indicating that the region upstream of ATIC transcription start site harbors a long non-coding RNA (lncRNA) ZFAS1 binding site. Region upstream of ATIC transcription start site cloned into the luciferase reporter vector. Cells co-transfected with si-NC and si-lncRNA ZFAS1. RT-PCR performed after transfection to detect luciferase activity. (**B**) The cancer genome atlas (TCGA) dataset revealed a positive correlation between lncRNA ZFAS1 and ATIC. (**C**) RT-PCR results of ATIC expressions in HepG2 cells treated with si-lncRNA ZFAS1. (**D**) Western blot results of ATIC expressions in HepG2 cells treated with si-lncRNA ZFAS1. (**E**) RT-PCR analysis of ATIC expression in HCC cells transfected with control (scrambled), si-ATIC-1, si-ATIC-2, and si-ATIC-3. (**F**) Western blot analysis of ATIC expression in HCC cells transfected with control (scrambled), si-ATIC. (**G**) CCK-8 assay results showing the viability of HCC cells treated with si-ATIC. (**H**) Colony formation assay results revealing the proliferation of si-ATIC-transfected HCC cells and colonies. (**I**) Transwell assay results revealing the migration of HCC cells treated with si-ATIC. (**J**) Wound healing assay results revealing the migration of HCC cells treated with si-ATIC. *P < 0.05, compared with Blank group. ^#^*P* < 0.05, compared with si-NC group

Analysis using the Miranda v3.3a software revealed a binding interaction between the upstream region of the ATIC gene transcription start site and lncRNA ZFAS1 (Table S1). To further explore this interaction, we cloned the region upstream of the ATIC transcription start site into a pGL3 luciferase vector and transfected it into si-NC and si-lncRNA cells. As depicted in Fig. 4A, the knockdown of ZFAS1 resulted in a decline in luciferase activity. TCGA database revealed a positive association between the expression of lncRNA ZFAS1 and ATIC in HCC tissues (Fig. 4B). Furthermore, RT-PCR and western blotting results demonstrated a notable reduction in ATIC expression in the si-lncRNA ZFAS1 group compared to the blank and si-NC groups (Fig. 4C, D). These findings indicate that lncRNA ZFAS1 can activate ATIC gene transcription.

Subsequently, ATIC knockdown experiments were performed to verify the role of ATIC in HCC development. As depicted in Fig. 4E, the expression of ATIC was significantly decreased in the siRNA-ATIC-1, siRNA-ATIC-2, and siRNA-ATIC-3 groups compared to that in the si-NC group. As siRNA-ATIC-1 had the highest knockout efficiency in HepG2 cells, siRNA-ATIC-1 was used for follow-up experiments. Western blotting revealed significantly lower ATIC expression in the si-ATIC group than in the blank and si-NC groups (Fig. 4F, *P* < 0.05). CCK-8 and colony formation assays revealed that si-ATIC significantly inhibited HCC cell proliferation compared to that in the blank and si-NC groups (Fig. 4F, G, *P* < 0.05). Furthermore, transwell migration and wound healing assays revealed that ATIC knockdown significantly inhibited the migration of HCC cells compared to the control and si-NC groups (Fig. 4H, I, *P* < 0.05). These results confirmed that knockdown of either ATIC or lncRNA ZFAS1 exerts a similar inhibitory effect on HCC proliferation and metastasis.

### LncRNA ZFAS1- ATIC axis regulated proliferation and migration via PI3K/AKT pathway

**Fig. 5:**
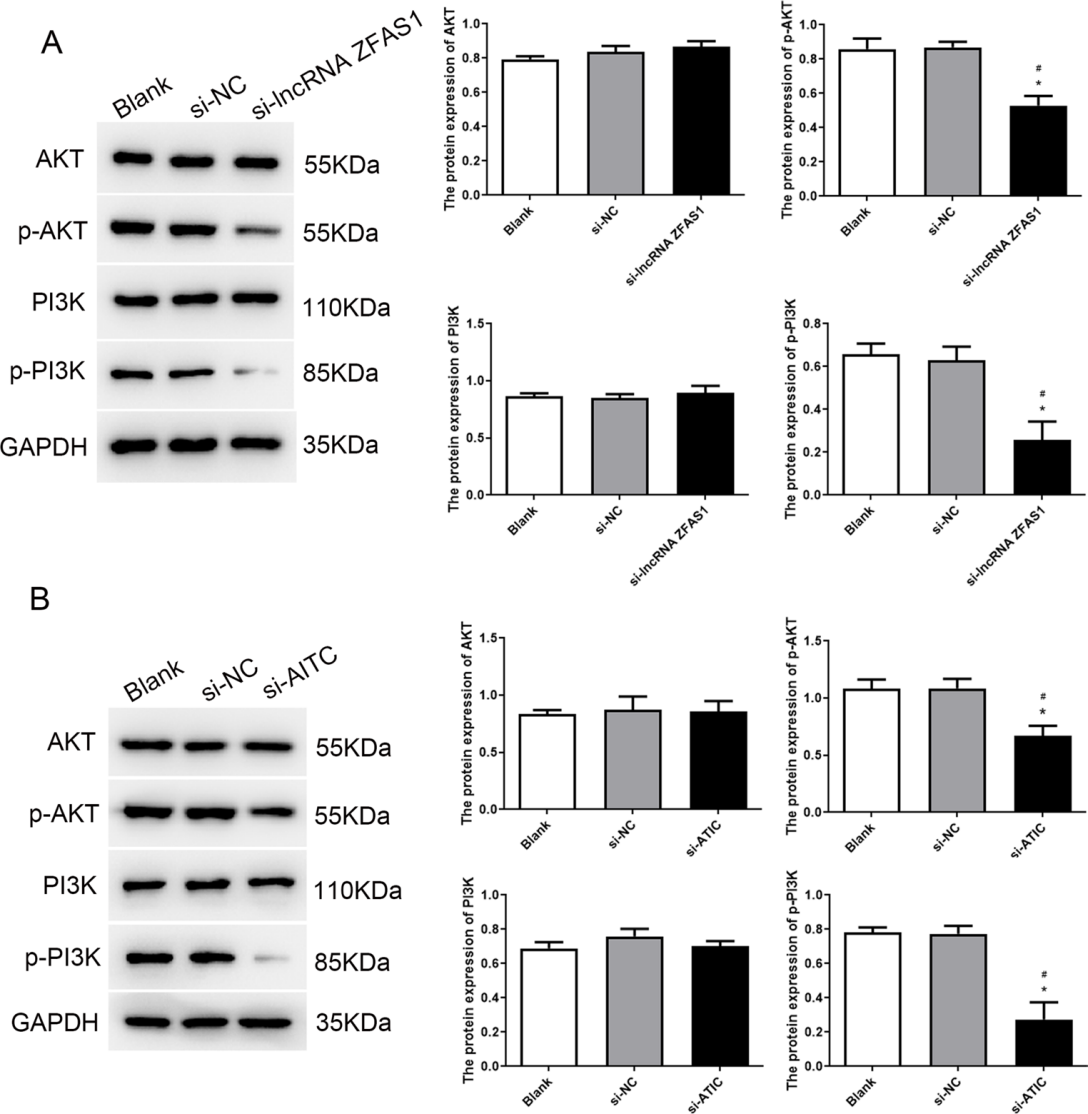
Western blot results of PI3K, p-PI3K, AKT, and p-AKT expressions in HepG2 cells treated with si-lncRNA ZFAS1 (**A**) and si-ATIC (**B**)

The PI3K/AKT pathway is widely recognized as a pivotal oncogenic pathway in diverse types of cancers, including HCC (Paskeh et al. 2023; Vara et al. 2004). Moreover, the PI3K/AKT pathway plays a crucial role in governing fundamental biological functions such as cell proliferation and metabolism. Therefore, we investigated the effect of the ZFAS1-ATIC axis on the PI3K/AKT pathway in HCC. To test this hypothesis, western blotting was performed to assess the protein levels of p-PI3K, PI3K, AKT, and p-AKT. Remarkably, our findings demonstrated that treatment with si-lncRNA ZFAS1 and si-ATIC resulted in decreased expression of p-PI3K and p-AKT compared to that in the blank and si-NC groups (Fig. 5).

**Fig. 6:**
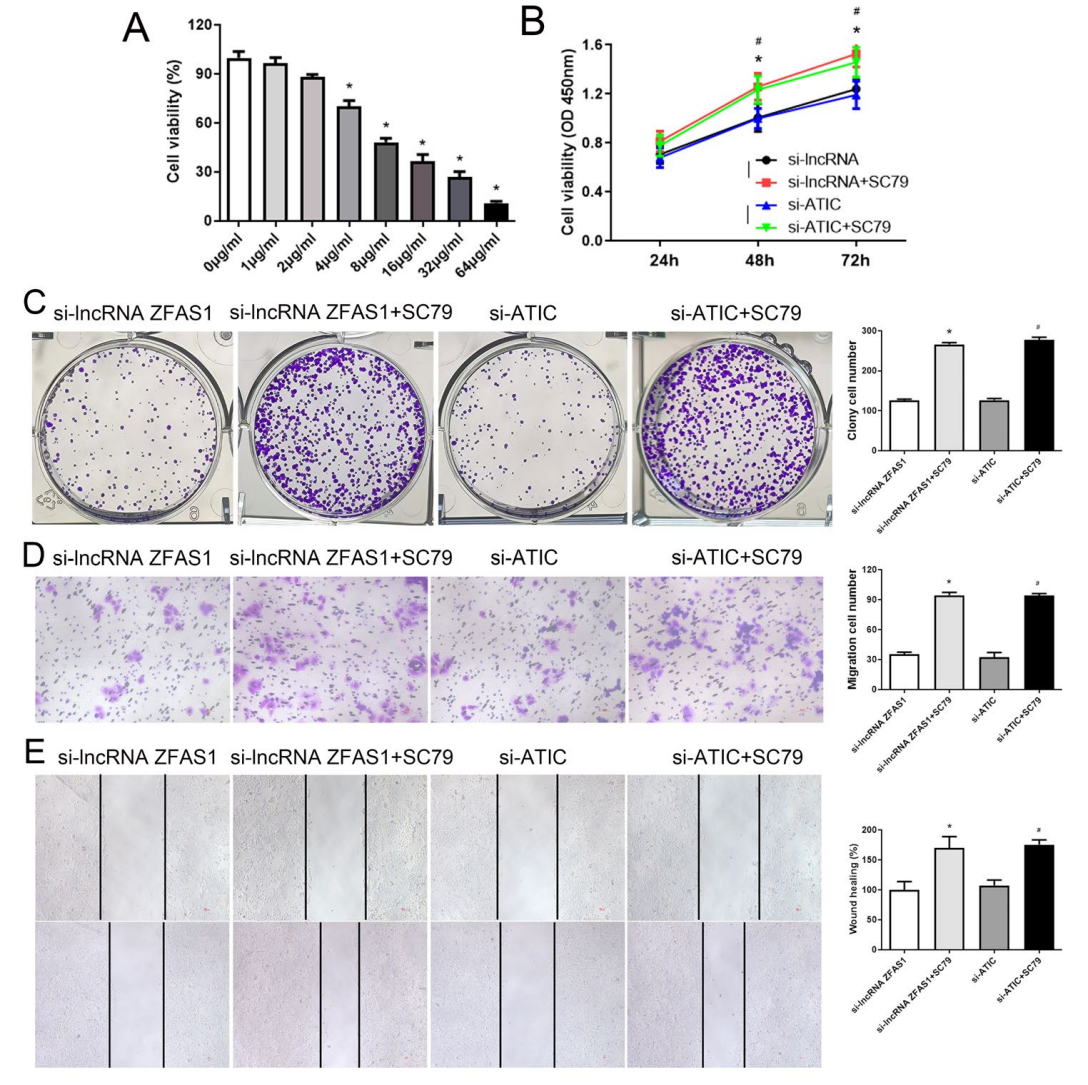
SC79 (a AKT activator) partially restores long non-coding RNA (lncRNA) ZFAS1 and ATIC knockdown HepG2 cells. (**A**) CCK-8 assay results revealing the viability of HCC cells treated with different concentration of SC79. (**B**) CCK-8 assay results revealing the viability of HCC cells treated with different treatments. (**C**) Colony formation assay results revealing the proliferation of HCC cells treated with different treatments and colonies were counted and captured. (**D**) Transwell assay results revealing the migration of HCC cells treated with different treatments. (**E**) Wound healing assay results revealing the migration of HCC cells treated with different treatments. *P < 0.05, compared with si-lncRNA ZFAS1. ^#^*P* < 0.05, compared with si-ATIC

To further investigate whether the lncRNA ZFAS1-ATIC axis regulates HCC migration and proliferation through the PI3K/AKT pathway, a series of rescue experiments were conducted using SC79, an AKT activator. Initially, we observed that cell proliferation was not inhibited with increasing concentrations of SC79 from 0 to 2 µg/mL (Fig. 6A). However, cell viability was induced in a concentration-dependent manner upon treatment with SC79 (4, 8, 16, 32, 64 µg/mL). Consequently, 2 µg/mL SC79 was utilized for further experiments. As illustrated in Fig. 6B, C, D, and E, CCK-8, colony formation, transwell migration, and wound healing assays revealed that the cells exhibited significantly augmented cellular proliferation and migration capabilities compared to the si-lncRNA ZFAS1 group. Similarly, the si-ATIC + SC79 group exhibited significantly enhanced cell proliferation and migration compared to the si-ATIC group (Fig. 6B, C, D, and E, *P* < 0.05). These findings indicate that the lncRNA ZFAS1-ATIC axis promotes HCC progression by modulating the PI3K/AKT pathway.

### Knockdown of lncRNA ZFAS1 and ATIC suppresses tumor growth and metastasis in nude mice

**Fig. 7:**
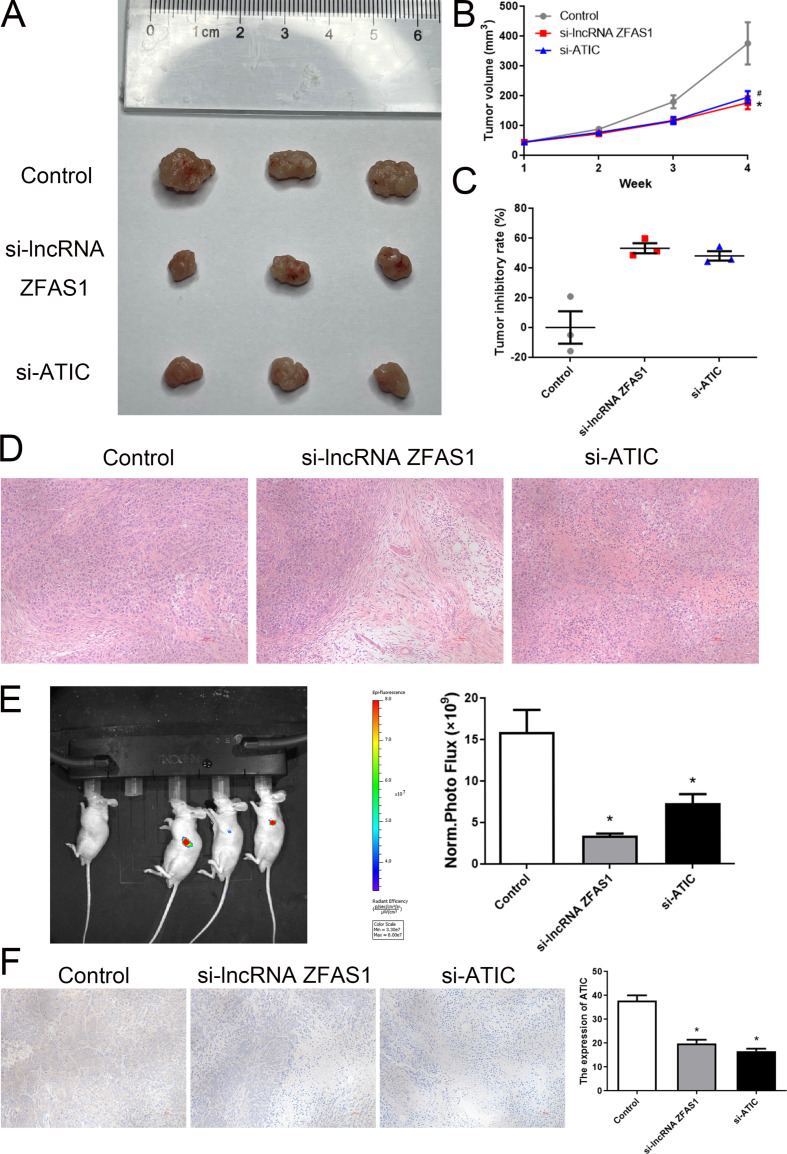
Knocking down lncRNA ZFAS1 and ATIC could inhibit the growth of HCC in vivo. (**A**) HepG2 cells transfected with si-lncRNA ZFAS1 or si-ATIC were subcutaneously injected into the armpit of nude mice (*n* = 3 each group). Tumors before and after carrying from the nude mice. (**B**) Tumor volumes calculated after injection every week. (**C**) Tumor inhibition rate of each group. (**D**) Tumor sections with hematoxylin and eosin (H&E) staining. (**E**) Representative image and analysis of luminescence intensity in the mice models. (**F**) Tumor sections with immunohistochemistry (IHC) staining. *P < 0.05, compared with control group

**Fig. 8:**
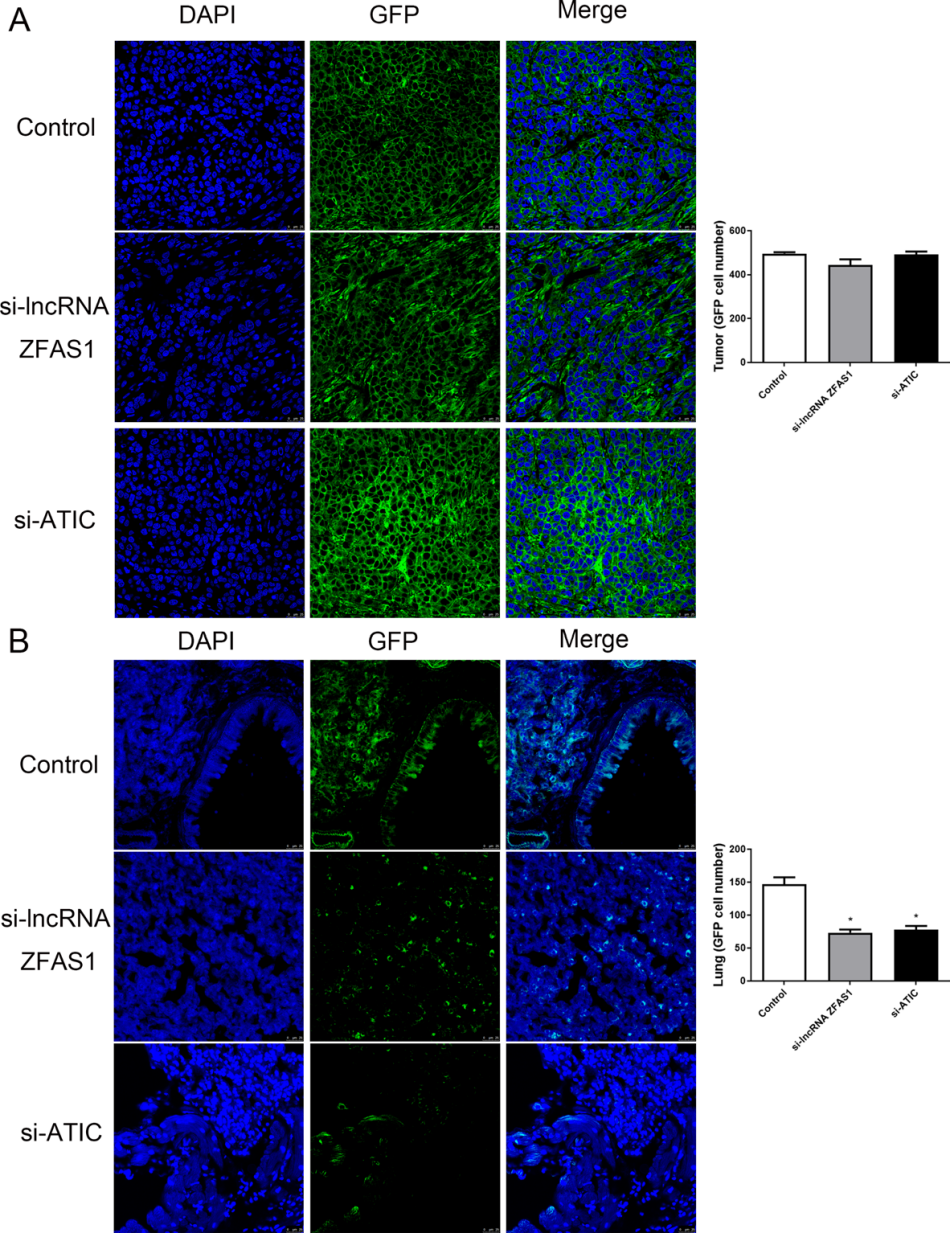
Immunofluorescence staining of tumor tissues (**A**) and lung tissues (**B**)

To validate the inhibitory effects of ZFAS1 and ATIC in vivo, HepG2 cells with si-lncRNA ZFAS1 or si-ATIC and injected into nude mice. Notably, Fig. 7A, B, C demonstrate a substantial reduction in tumor volume in the si-lncRNA ZFAS1 and si-ATIC groups, in contrast to the control group, after 4-week. The In Vivo Imaging System detected a significant decrease in the intensity of green fluorescence emitted by the si-lncRNA ZFAS1 and si-ATIC groups compared to that in the control group (Fig. 7D). Morphological alterations were observed in the si-lncRNA ZFAS1, si-ATIC, and control groups by histological examination using HE staining (Fig. 7E). IHC staining revealed decreased ATIC expression in the si-lncRNA ZFAS1 and si-ATIC groups relative to the control group (Fig. 7F, *P* < 0.05). Moreover, the immunofluorescence results revealed no significant differences in the number of green fluorescent cells in tumor tissues between the si-lncRNA ZFAS1 group and si-ATIC group compared to that in the control group (Fig. 8A, *P* > 0.05). However, the number of green fluorescent cells was significantly diminished in the lung tissues of both the si-lncRNA ZFAS1 and si-ATIC groups compared to that in the control group (Fig. 8B, *P* < 0.05).

## Discussion

**Fig. 9:**
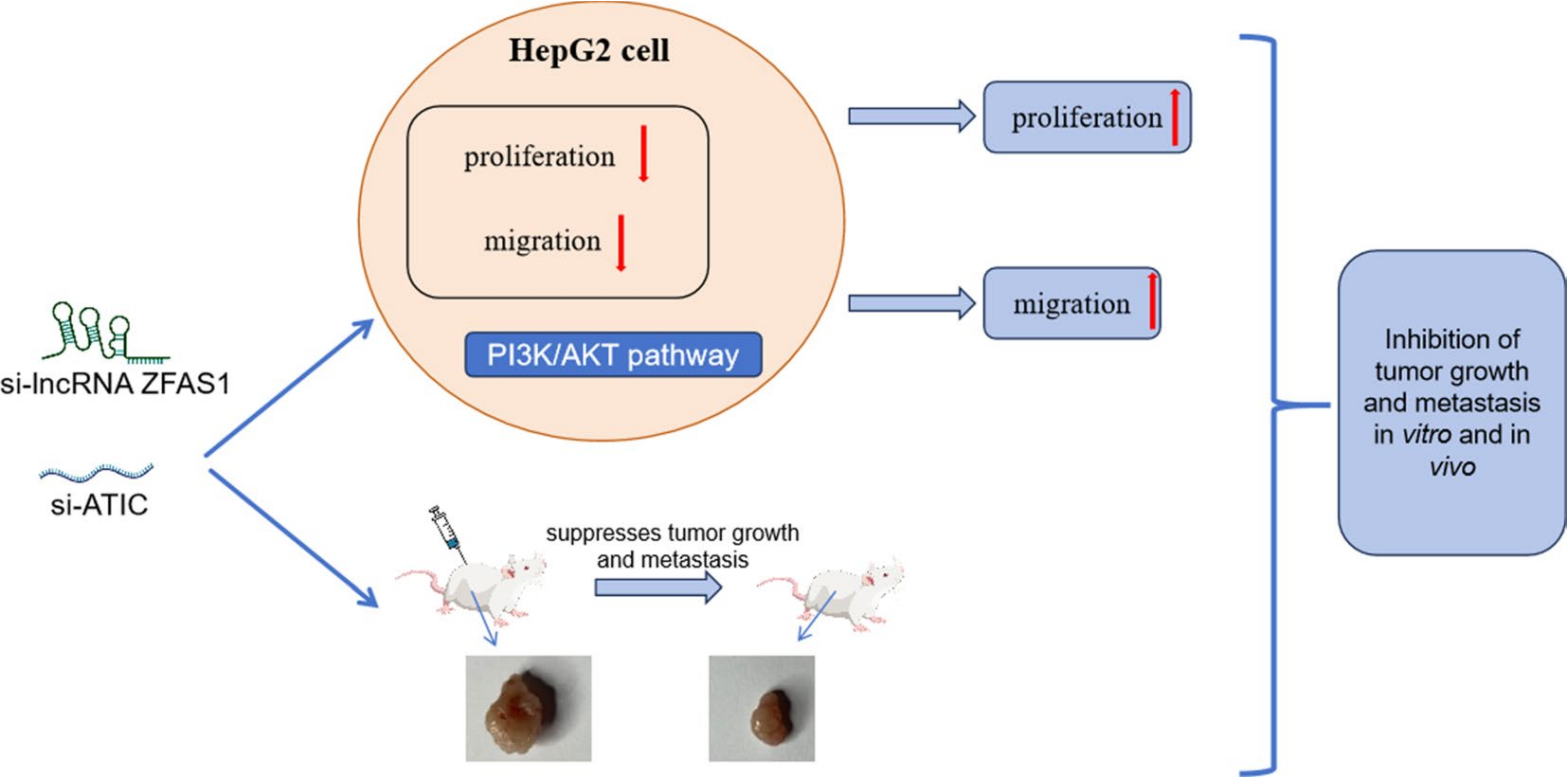
Regulatory mechanism of LncRNA ZFAS1 in modulating ATIC transcription and promoting the growth and migration of HCC through the PI3K/AKT signaling pathway is elucidated. In in vitro experiments showing the inhibition of HepG2 cell proliferation and migration upon the knockdown of si-lncRNA ZFAS1 and si-ATIC, which was reversed upon the addition of an AKT activator. In vivo experiments showed that si-lncRNA ZFAS1 and si-ATIC could inhibit tumor growth and lung metastasis

HCC is a widely recognized malignancy worldwide and is the third most prevalent cause of cancer-associated mortality. HCC is the main histological subtype of primary liver cancers and accounts for 70% of the worldwide burden of liver cancer (Jemal et al. 2011; Perz et al. 2006). The insidious onset and lack of early clinical symptoms make the early detection and diagnosis of HCC challenging (Zong et al. 2020). Typically, HCC is diagnosed at advanced stages, contributing to its persistently high mortality rates owing to its propensity for invasion and metastasis (Chidambaranathan-Reghupaty et al. 2021). Consequently, there is an urgent need to identify valuable molecular markers for the early detection and identification of therapeutic targets for the treatment of HCC. In this study, we revealed that lncRNA ZFAS1 and ATIC were highly expressed in HCC tissues, and that lncRNA ZFAS1 could directly bind to ATIC to form the lncRNA ZFAS1-ATIC axis. Furthermore, the knockdown of lncRNA ZFAS1 and ATIC markedly suppressed the growth and migratory capabilities of HCC cells. Further mechanistic studies showed that the ZFAS1-ATIC axis regulates the biological role of HCC cells by activating the PI3K/AKT signaling pathway. Furthermore, we verified that the knockdown of ZFAS1 and ATIC inhibited HCC growth in vivo in a nude mouse transplantation model. In summary, our study revealed the important role and molecular mechanism of the lncRNA ZFAS1-ATIC axis in HCC, providing new targets and strategies for the treatment of HCC (Fig. 9).

LncRNAs, a distinct category of non-coding RNAs, exert a crucial influence on gene expression modulation through diverse mechanisms, including chromatin structure remodeling (Huang et al. 2021b), DNA methylation regulation, and modulation of transcriptional activation or interference. ZFAS1, an intriguing lncRNA transcribed in the opposite orientation to the NFX1 zinc finger gene, resides on the 20q13.13 locus of the human chromosome. Initially, ZFAS1, a novel tumor-associated lncRNA, was found to enhance epithelial-mesenchymal transition, migration, and cell proliferation (Askarian-Amiri et al. 2011). Recently, the involvement of LncRNA-ZFAS1 in the pathogenesis of various cancers has been extensively reported (Chen et al. 2018; Pan et al. 2020; Xu et al. 2018). For instance, Zhang et al. showed that lncRNA ZFAS1 curtails the migration, invasion, and growth of breast cancer cells by modulating the PTEN/PI3K/AKT pathway via its interaction with miR-589 (Zhang et al. 2020). Liu et al. demonstrated that ZFAS1 promotes metastasis in pancreatic adenocarcinoma through the RHOA/ROCK2 pathway by acting as a miR-3924 sponge (Liu et al. 2020). In HCC, ZFAS1 is an lncRNA capable of influencing disease progression through diverse regulatory pathways. Zhang et al. substantiated the direct interaction between lncRNA ZFAS1 and miR-150-5p, which exerts a profound influence on the migration, proliferation, and invasion of HCC cells (Zhu et al. 2023b). Similarly, the study conducted by Duan et al. demonstrated that the depletion of lncRNA ZFAS1 hinders the progression of HCC by suppressing the ERK/MDK/JNK/P38 pathway and restoring the expression of miR-624 (Duan et al. 2020a). Furthermore, Zhou et al. showed that the suppression of lncRNA ZFAS1 significantly curtailed the proliferation of HCC cells, whereas its overexpression of lncRNA ZFAS1 fosters their proliferation (Zhou et al. 2019). Consistent with previous findings, our study revealed increased expression of lncRNA ZFAS1 in HCC and highlighted the inhibitory effects of si-ZFAS1 on the growth and migration of HCC cells.

ATIC is a bifunctional enzyme with a molecular weight of 64 kDa that regulates the activities of two enzymes involved in de novo purine biosynthesis, IMP cyclohydrolase and AICAR (Greasley et al. 2001). There is a correlation between ATIC and rheumatoid arthritis, tumor cell proliferation, and the effectiveness of radiotherapy (Liu et al. 2018; Lv et al. 2018). Niu et al. revealed a noteworthy upregulation of ATIC in the tissues and cells of lung adenocarcinoma, and this increased expression exhibited a strong correlation with diminished survival rates and an advanced clinical stage among LUAD patients (Niu et al. 2022). A comprehensive in vitro and in vivo study demonstrated that ATIC facilitates the development of liver cancer via the AKT/FOXO3 pathway (Zhang et al. 2021). Additionally, Li et al. provided evidence of an anomalous elevation of ATIC expression in HCC tissues, and knockdown of ATIC expression yielded a conspicuous reduction in the colony formation, proliferation, and migration capabilities of HCC cells (Li et al. 2017). In the present study, we elucidated a direct interaction between ATIC and the lncRNA ZFAS1, and the knockdown of ATIC led to the suppression of proliferation and migration in HepG2 cells. Additionally, in vivo research has demonstrated that knockdown of both ATIC and lncRNA ZFAS1 can inhibit tumor growth and lung metastasis. These findings highlight the involvement of ATIC in the functional modulation of HCC cells through the mediation of lncRNA ZFAS1.

The PI3K/AKT pathway is a critical intercellular signaling cascade that is pivotal in driving cancer progression, metastasis, and metabolic alterations (Huang et al. 2021a). The PI3K/AKT pathway has been known to be activated in various tumors, including HCC (Wu et al. 2020). Recent studies have shed light on the role of lncRNAs in regulating the PI3K/AKT pathway. For example, Wang et al. discovered that lncRNA ZFAS1 stimulates proliferation and migration while suppressing apoptosis in nasopharyngeal cancer via the activation of the PI3K/AKT pathway (Wang et al. 2019b). Similarly, Wang et al. proposed that the upregulation of the lncRNA FER1L4 exerts a suppressive effect on the migration and proliferation of HCC cells by modulating the PI3K/AKT signaling pathway (Wang et al. 2019a). Furthermore, Zheng et al. found that LINC01133 knockdown significantly impedes HCC tumor development by targeting the PI3K/AKT pathway (Zheng et al. 2019). SC79 functions as an activator of AKT, eliciting the phosphorylation of AKT to initiate activation of the PI3K/AKT signaling cascade (Luan et al. 2018). Here, we observed that SC79 partially restored the suppressive consequences of ZFAS1/ATIC downregulation on HCC cell proliferation and migration. Consequently, we deduced that the ZFAS1-ATIC axis promotes HCC cell proliferation and migration by modulating the PI3K/AKT signaling pathway.

However, this study had some limitations. First, we only examined the ceRNA network that regulates lncRNA ZFAS1 in ATIC, while the other regulatory mechanisms mentioned above that may be involved require further investigation. Second, the specific miRNAs involved in the ZFAS1-ATIC axis have not been identified, and the potential upstream regulatory factors controlling this axis have not been explored. Therefore, further identification of specific miRNAs involved in the ZFAS1-ATIC axis and exploration of potential upstream regulatory factors controlling this axis is required.

In summary, we uncovered the pivotal significance of the lncRNAs ZFAS1 and ATIC in the progression of HCC. Our findings demonstrate that lncRNA ZFAS1 regulates ATIC transcription and promotes the proliferation and migration of HCC cells through the PI3K/AKT pathway. Moreover, the lncRNA ZFAS1-ATIC axis holds promise as both a diagnostic biomarker and therapeutic target for HCC.

## Electronic supplementary material

Below is the link to the electronic supplementary material.

Supplementary Material 1

## Data Availability

Data is provided within the manuscript or supplementary information files. The authors confirm that the data supporting the findings of this study are available within the article.
